# Rational Therapeutics of Cholera Infantum*Reprint from *The New York Medical Journal*, March 2, 1895.

**Published:** 1895-06

**Authors:** Gustavus Blech

**Affiliations:** St. Louis; 11 N. Broadway


### Selections and Abstracts.

---

### RATIONAL THERAPEUTICS OF CHOLERA INFANTUM.*

By GUSTAVUS BLECH, M. D., St. Louis.

No strict rules can be given for the treatment of disease. It is for this reason that so many physicians say we do not treat a disease, but we treat an individual. True enough, we treat the individual, but what we have most of all to consider is the disease. The individual will dictate us alterations and modifications in our treatment.

A general plan of treatment may be outlined, however, and I will try to do so in regard to one of the most fatal diseases of babyhood—cholera infantum. There is a certain philosophy in therapeutics which I would frame in the three following rules: First, remove if possible the disturbing causes; second, treat symptoms which *per se* are liable to endanger the life of the patient; and third, sustain vitality.

As said before, the therapeutics, which is based upon the ætiology and pathology of a given case is the only one to be employed.

Now, the ætiology of cholera infantum is not so obscure as asserted by a good many authors. Whether or not of microbic origin, one thing is sure—it is due to a chemical decomposition of food, causing an inflammatory condition of the digestive and alimentary canal.

Clinical experience, furthermore, shows that this disease is of a grave character, producing death in a large proportion. Heat *per se* is not the immediate cause of this disease, but it influences its course considerably. Therefore, gastric or intestinal disturbances in summer demand a closer attention than those which occur during the colder season. Cholera infantum is a disease met even in the palaces of the rich, although not so often as in the tenement houses of the poor, which fact proves again that bad air, filth and lack of ventilation are also of a predisposing influence, as well as an obstacle to a quick cure. The mortality in the tenement houses is larger than that of the richer parts.

If we consider the aforesaid, we shall first of all, as regards the treatment of this disease, have to restrict diet.

As soon as called to a case of cholera infantum, prohibit for the first day any food whatever. Mothers have no right to nurse the little patient either. Strict instructions must be

---

*Reprint from *The New York Medical Journal*, March 2, 1895.

given in that direction, because the timid mothers are often inclined to quiet the crying babies by putting them to the breast.

Remedies are of very little value. Beginning with calomel, salol, and all the newer antiseptics, finishing with subnitrate of bismuth—they have all proved a failure, for none of them work quickly enough.

The treatment as outlined by Dr. Elmer Lee, of Chicago, in his cases of typhoid fever, proved a success in my hands during last summer, and under this treatment I have lost only one patient out of twenty-three, while the monuments of my skill exercised during the year 1893 are decorating the cemeteries of the State of Connecticut.

So far as I knew, the best antiseptic (which has also a strong tendency to reduce local inflammation) was peroxide of hydrogen (medicinal) until hydrozone was used by me. Hydrozone being twice as strong as Marchand's peroxide of hydrogen (for ecomical reasons), the latter drug is preferred by me. This remedy can be administered internally as well as externally.

I add a tablespoonful of hydrozone to a pint of water for washing out the stomach. The vomiting ceases after the first washing as a rule. If necessary, this procedure can be repeated. If the vital power of the little patient is not too low it can produce no harm. But in every case, no matter how far advanced, I do not omit an irrigation of the bowels, for which purpose I use a soft rubber catheter attached to a common bulb syringe. The catheter is introduced as high in the colon as possible. It is unnecessary to say that the water must first be sterilized. I do not agree with Dr. Lee in using hot soap water. On the contrary, I use cold water, and add to each quart about two ounces of hydrozone. The improvement after the first or second irrigation is marked. If necessary, these irrigations can be repeated every two hours.

Among other remedies there are only two to be employed. morphine and strychnine. Both ought to be administered hypodermically. Their indication is too well-known and they are about all we need. No antipyretics should be given. If the fever is very high and if the irrigation of the bowels does not reduce it, the whole body should be washed with alcohol.

The diet for the next twenty-four hours should be very light indeed. Sweet, strong Russian tea is all I allow.

Each individual case will teach us when food can be allowed again.

Since the adoption of this mode of treatment I have met with the most remarkable success, and no honest practitioner should refuse it a trial.

*11 N. Broadway.*

